# Therapeutics of Nervous Diseases with Their Diagnosis and Pathology

**Published:** 1889-12

**Authors:** 


### Therapeutics of Nervous Diseases, with their Diagnosis and Pathology. By Charles Porter Hart, M. D. Hahnemann Publishing House, Philadelphia.

All of the more common diseases classified as "nervous diseases" are discussed in Professor Hart's book. We find a brief description of each disease with mention of the remedies most frequently called for in each case. In all the varied range of medical practice there is no class of subjects more difficult to treat of than the so-called nervous diseases. Dr. Hart's work will be found of use to the student in studying these diseases. The volume is published in the usual neat form which characterizes the work of the Hahnemann Publishing House. Price is $2.00.

